# Modeling and validation in Parkinson’s disease patients with frailty

**DOI:** 10.3389/fnins.2025.1723707

**Published:** 2025-12-08

**Authors:** Guoyang Li, Guo Hong, Jing Huang, Wenli Zhang, Fengju Mao, Xiaoguang Luo

**Affiliations:** 1 Department of Neurology, Second Clinical Medical College of Jinan University, Shenzhen People’s Hospital, Guangdong, China; 2 The First Affiliated Hospital, Southern University of Science and Technology, Shenzhen, Guangdong, China; 3 Shenzhen Clinical Research Centre for Geriatrics, Shenzhen People’s Hospital, Shenzhen, Guangdong, China; 4 Department of Geriatrics, Shenzhen Second People's Hospital, Shenzhen, Guangdong Province, China

**Keywords:** Parkinson’s disease, frailty, machine learning, early screening, MOCA, HAMD, HAMA

## Abstract

**Introduction:**

Parkinson’s disease (PD) is the second most common neurodegenerative disorder. The risk of frailty is significantly higher in patients with PD than in age-matched individuals without PD. This study aimed to develop a machine learning–based predictive model for frailty in PD.

**Methods:**

We conducted a cross-sectional study of early- and middle-stage PD patients recruited from June 2024 to June 2025 at Shenzhen People’s Hospital. Frailty was assessed using the Fried criteria (five components: gait speed, grip strength, physical activity, fatigue, and weight loss). A total of 42 demographic and clinical variables, including disease history, Montreal cognitive assessment (MoCA), and unified Parkinson’s disease rating scale (MDS-UPDRS) scores, were collected and compared between PD patients with and without frailty. Spearman correlation and LASSO regression were used to identify independent risk factors. Multiple machine learning algorithms were applied to construct predictive models. Model performance was evaluated using receiver operating characteristic (ROC) curves, area under the ROC curve (AUC), decision curve analysis (DCA), calibration plots, and forest plots.

**Results:**

A total of 205 PD patients were enrolled (133 non-frail, 72 frail; mean age non-frail 62.92 ± 9.69 years, frail 68.13 ± 8.44 years). Significant group differences were found in sex (*p* = 0.013), age (*p* < 0.001), disease severity (MDS-UPDRS, *p* < 0.001; modified Hoehn-Yahr stage (H&Y stage), *p* < 0.001), alcohol consumption (*p* = 0.010), MoCA (*p* < 0.001), HAMD (*p* = 0.001), and Hamilton anxiety rating scale (HAMA) (*p* < 0.001). Eight features were identified as independent predictors of frailty: sex, age, alcohol use, Modified H&Y stage, UPDRS-IV score, HAMA score, executive function, and naming. Among all tested algorithms, logistic regression achieved the best predictive performance (AUC = 0.83 in the test set), outperforming other machine learning models.

**Conclusion:**

Frailty in PD was associated with female sex, older age, alcohol use, and more advanced disease severity. Patients with PD and frailty exhibited higher MDS-UPDRS scores, more severe cognitive impairment, and greater levels of depression and anxiety. Integrating clinical data with machine learning, especially logistic regression, provides a reliable and scalable tool for early identification and risk stratification of frailty in PD.

## 1 Introduction

Parkinson’s disease (PD) is a progressive neurodegenerative disorder and the second most prevalent age-related neurological disease. Among all neurological disorders, PD shows the most rapid growth in prevalence, mortality, and disability-adjusted life years worldwide (Bloem et al., 2021; Liu et al., 2025). PD is characterized by a spectrum of motor and non-motor symptoms, reflecting its complex, heterogeneous, and progressive nature. Key features include motor disturbances such as tremor, rigidity, bradykinesia, and postural instability, as well as non-motor manifestations like hyposmia and sleep disturbances (Simon et al., 2020; Bloem et al., 2021). PD is characterized by the degeneration and loss of dopaminergic neurons in the substantia nigra pars compacta, which projects to the striatum, and by the presence of Lewy bodies (Balestrino and Schapira, 2020).

Frailty is a geriatric syndrome characterized by diminished physiological reserve and impaired resilience across multiple physiologic systems. This heightened vulnerability to stressors predisposes individuals to adverse health outcomes (Fried et al., 2001; Thillainadesan et al., 2020). Frailty, as defined by Fried and colleagues using five specific criteria (slow gait speed, reduced grip strength, low physical activity, fatigue, and weight loss), is highly prevalent among older adults. Its prevalence ranges from 12–24% for frailty and 46–49% for pre-frailty, depending on the assessment tool and population. This condition is strongly associated with numerous adverse outcomes, including falls, fractures, disability, dementia, dependency, hospitalization, long-term care needs, and mortality (Satake and Arai, 2020; O’Caoimh et al., 2021; Karway et al., 2025). Importantly, the prevalence of frailty is markedly higher in PD patients (29–67%) than in age-matched older adults without PD (approximately 10%) (Ahmed et al., 2008; Collard et al., 2012; Roland et al., 2012; Liotta et al., 2017; Renne and Gobbens, 2018).

In recent years, machine learning (ML) has emerged as a transformative tool in neurology and other biomedical fields, demonstrating significant value in enhancing diagnostic accuracy, informing decision-making, and advancing risk prediction, disease diagnosis, and personalized therapeutic strategies (Haug and Drazen, 2023; Oikonomou and Khera, 2023; Zhang et al., 2024; Cai et al., 2025). Common ML algorithms, including logistic regression (LR), linear discriminant analysis (LDA), k-nearest neighbors (KNN), random forest (RF), gradient boosting machines (GBM), support vector machines (SVM), naïve Bayes (NB), and artificial neural networks (ANN; Desai et al., 2020; Hsiung et al., 2023), have demonstrated utility in predicting frailty among older adults, hemodialysis patients, and those with cardiovascular or metabolic diseases (Deberneh and Kim, 2021; Leghissa et al., 2023, 2024; Lv et al., 2024; Zhang et al., 2024; Cao et al., 2025). However, to date, no studies have developed ML-based models specifically for predicting frailty in PD.

Given the clinical importance of early identification and intervention, developing a simple, accurate, and scalable frailty prediction model in PD may not only improve patient outcomes but also reduce healthcare burden. Therefore, this study aimed to: (1) examine the relationships between demographic and clinical features (e.g., MDS-UPDRS, cognitive, anxiety, and depression assessments) and frailty in PD; and (2) develop and validate an ML-based predictive model for frailty in PD as a practical screening tool.

## 2 Methods

### 2.1 Study design and participants

This cross-sectional study was conducted at Shenzhen People’s Hospital from June 2024 to June 2025. Consecutive patients with idiopathic Parkinson’s disease (PD) were enrolled.

PD was diagnosed according to the 2015 Movement Disorder Society (MDS) Clinical Diagnostic Criteria for PD (Postuma et al., 2015). Inclusion Criteria: (1) Definite diagnosis of idiopathic Parkinson’s disease (PD), confirmed by attending neurologists specializing in movement disorders according to the MDS Clinical Diagnostic Criteria for Parkinson’s Disease (2015), with secondary parkinsonism excluded; (2) Modified Hoehn & Yahr (H&Y) stage 1–3, determined through in-person motor function assessments; (3) Age between 18 and 80 years, verified using official identification documents (e.g., ID card); (4) Capacity to complete clinical evaluations and provide informed consent: participants were required to communicate coherently, comprehend study procedures, complete neuropsychological tests (e.g., MoCA, HAMD, HAMA), and perform physical assessments (e.g., gait speed, grip strength). For patients with mild cognitive impairment, informed consent was obtained from both the patient and their legal guardian after confirming the patient’s understanding of the study objectives. Exclusion Criteria: (1) Comorbid neurological or psychiatric conditions (e.g., Alzheimer’s disease, multiple system atrophy, stroke, traumatic brain injury, schizophrenia, bipolar disorder, or major depressive disorder); (2) Severe systemic diseases: active malignancy (diagnosed within the past 5 years), end-stage cardiac, hepatic, or renal disease, uncontrolled hypertension (systolic BP > 180 mmHg or diastolic BP > 110 mmHg despite medication), or acute infections (e.g., pneumonia, sepsis) within 1 month prior to enrollment; (3) Secondary parkinsonism resulting from medications (e.g., antipsychotics, metoclopramide), vascular lesions (confirmed by brain MRI), toxins, or metabolic disorders (e.g., Wilson’s disease); (4) Incomplete clinical data necessary for model construction and validation (e.g., missing Fried frailty criteria assessments or key variables such as age, sex, or MDS-UPDRS scores).

Patients were consecutively recruited from the Movement Disorders Clinic at Shenzhen People’s Hospital. Eligibility was initially screened by neurologists through medical record review and clinical interviews. After obtaining informed consent, participants underwent comprehensive assessments, including: Collection of demographic data (age, sex, education level, medical history, alcohol use, etc.); Motor function evaluation using the MDS-UPDRS and Modified H&Y staging; Cognitive assessment via the MoCA; Evaluation of emotional state using the HAMD and HAMA; Frailty assessment based on Fried criteria (gait speed, grip strength, physical activity, fatigue, and weight loss). All assessments were conducted by trained researchers who were blinded to the study hypotheses to minimize potential bias. A total of 42 demographic and clinical variables were collected, including age, sex, disease duration, education level, smoking and alcohol consumption, comorbidities, and Levodopa equivalent daily dose (LEED) (Jost et al., 2023). Smokers were identified using the question “Have they smoked at least one cigarette per day for the past year or longer?” (Tan et al., 2008). Any drinker was defined as “a person who consumes at least one glass alcoholic beverage per week.” Weekly pure alcohol intake was subsequently calculated. Participants were classified as drinkers if they had a history of alcohol consumption during the pre-Parkinson’s disease (PD) onset period and consumed, on average, at least one standard drink per week. Individuals who did not meet this criterion were classified as non-drinkers (Ragonese et al., 2003). Alcohol consumption status was determined using the questions: “Have you ever consumed alcohol?” and “If yes, what types of alcoholic beverages do you typically consume per week, and what is your average alcohol intake?” Information was collected separately for each type of alcoholic beverage (e.g., beer, white wine, red wine, spirits). The amount of each beverage was converted to grams of pure alcohol based on standard conversion factors (Leone et al., 1997). One standard drink was defined as containing 0.6 fluid ounces (14 grams) of pure alcohol, equivalent to 12 fluid ounces (approximately 355 mL) of beer with 5% alcohol by volume (ABV), 5 fluid ounces (approximately 142 mL) of wine with 12% ABV, or 1.5 fluid ounces (approximately 44 mL) of spirits with 40% ABV (Alcohol Research: Current Reviews Editorial Staff, 2018). To determine whether a participant met the definition of a drinker, the total weekly intake of pure alcohol (in grams) was calculated by summing the alcohol content from all beverage types. Comorbidities included a history of hypertension, a history of atrial fibrillation (AF), a history of coronary heart disease (CHD), diabetes, hyperlipidemia, and other medical histories. Motor and non-motor symptoms were assessed using the following scales: MDS-Unified Parkinson’s Disease Rating Scale (MDS-UPDRS I–IV) (Chialà et al., 2018), Modified Hoehn & Yahr stage, Hamilton Depression Rating Scale (HAMD), Hamilton Anxiety Rating Scale (HAMA) and Montreal Cognitive Assessment (MoCA). The HAMA and HAMD are scales that have been recognized by previous expert groups as suitable for screening depression in patients with Parkinson’s disease (Leentjens et al., 2008; Torbey et al., 2015). MoCA subscores (Nasreddine et al., 2005) (executive function, naming, attention, language, abstraction, memory, and orientation) were also recorded.

### 2.2 Frailty assessment

Frailty status was evaluated using the Fried phenotype criteria, which comprise five components: (1) slowness, defined as gait speed < 0.8 m/s;(2) weakness, defined as grip strength below sex- and BMI-adjusted cutoffs;(3) low physical activity, assessed using the International Physical Activity Questionnaire (IPAQ);(4) self-reported fatigue; and (5) unintentional weight loss (>5% within the past year). Participants meeting three or more criteria were classified as frail, those with one or two criteria as pre-frail, and those with none as robust. In this study, the frail group was compared against the non-frail group (pre-frail + robust) (Fried et al., 2001).

### 2.3 Statistical analysis

All statistical analyses were performed using R software (version 4.3.2). Continuous variables were expressed as mean ± standard deviation (SD) or median (interquartile range, IQR), depending on distribution, and compared using independent-samples t test or Mann–Whitney U test. Categorical variables were expressed as frequencies (%) and compared using chi-square or Fisher’s exact test. Feature selection was conducted in two steps: (1) Correlation analysis: Spearman correlation coefficients were used to evaluate associations between variables and frailty status; (2) LASSO regression: The least absolute shrinkage and selection operator (LASSO) with 10-fold cross-validation was applied to identify independent predictors of frailty. A two-sided *p* value <0.05 was considered statistically significant.

### 2.4 Machine learning models

Based on selected features, multiple machine learning models were constructed, including logistic regression (LR), linear discriminant analysis (LDA), k-nearest neighbors (KNN), random forest (RF), gradient boosting machine (GBM), support vector machine (SVM), naïve Bayes (NB), and artificial neural network (ANN). Model performance was assessed in both training and test sets (70:30 split) using the following metrics: Receiver operating characteristic (ROC); curves and area under the curve (AUC); Calibration plots; Decision curve analysis (DCA); Forest plots of feature importance.

## 3 Results

As shown in Table 1, patients included in this study were individuals diagnosed with Parkinson’s disease (PD) who attended the PD Specialist Outpatient Clinic of Shenzhen People’s Hospital (the Second Clinical Hospital of Jinan University and the First Affiliated Hospital of Southern University of Science and Technology) between June 2024 and June 2025. Demographic and clinical data of 205 PD patients were collected, encompassing 42 clinically relevant variables, including demographic characteristics, medical history, frailty assessment criteria, cognitive function scores, and anxiety/depression scale scores.

**Table 1:** Baseline characteristics of participants.

Characteristics	Category item	PD (*n* = 205)	Non-frailty (*n* = 133)	Frailty (*n* = 72)	Z	*p*
Age, (years, mean ± SD)		64.75 ± 9.60	62.92 ± 9.69	68.13 ± 8.44	−3.82	<0.001
Sex, *n* (%)	Male	101 (49.27)	74 (55.64)	27 (37.50)	6.15	0.013
	Female	104 (50.73)	59 (44.36)	45 (62.50)		
Disease duration (years, mean ± SD)		4.39 ± 3.59	4.32 ± 3.51	4.51 ± 3.73	−0.36	0.723
Modified H&Y, (mean ± SD)		1.80 ± 0.71	1.60 ± 0.65	2.15 ± 0.68	−5.68	<0.001
LEDD (mg, mean ± SD)		456.80 ± 264.91	444.13 ± 256.73	480.21 ± 277.88	−0.93	0.354
Stage, *n* (%)	Early	170 (82.93)	121 (90.98)	49 (68.06)	17.33	<0.001
	Later	35 (17.07)	12 (9.02)	23 (31.94)		
Tape, *n* (%)	Tremor	20 (9.76)	16 (12.03)	4 (5.56)	2.63	0.268
	Spasms	68 (33.17)	45 (33.83)	23 (31.94)		
	Mix	117 (57.07)	72 (54.14)	45 (62.50)		
Diabetes mellitus, *n* (%)	No	197 (96.10)	129 (96.99)	68 (94.44)	0.81	0.369
	Yes	8 (3.90)	4 (3.01)	4 (5.56)		
Hypertension, *n* (%)	No	183 (89.27)	119 (89.47)	64 (88.89)	0.02	0.897
	Yes	22 (10.73)	14 (10.53)	8 (11.11)		
CHD, *n* (%)	No	195 (95.12)	125 (93.98)	70 (97.22)	1.06	0.304
	Yes	10 (4.88)	8 (6.02)	2 (2.78)		
Stroke, *n* (%)	No	199 (97.07)	130 (97.74)	69 (95.83)	0.6	0.438
	Yes	6 (2.93)	3 (2.26)	3 (4.17)		
Number of Chronic Diseases, (mean ± SD)		0.22 ± 0.56	0.22 ± 0.57	0.22 ± 0.56	−0.05	0.96
Smoking, *n* (%)	No	187 (91.22)	124 (93.23)	63 (87.50)	1.92	0.166
	Yes	18 (8.78)	9 (6.77)	9 (12.50)		
Alcohol drinking, *n* (%)	No	179 (87.32)	122 (91.73)	57 (79.17)	6.66	0.01
	Yes	26 (12.68)	11 (8.27)	15 (20.83)		
Tea, *n* (%)	No	189 (92.20)	122 (91.73)	67 (93.06)	0.11	0.735
	Yes	16 (7.80)	11 (8.27)	5 (6.94)		
Coffee, *n* (%)	No	202 (98.54)	131 (98.50)	71 (98.61)	0	0.948
	Yes	3 (1.46)	2 (1.50)	1 (1.39)		
Toxic exposure, *n* (%)	No	193 (94.15)	126 (94.74)	67 (93.06)	0.24	0.624
	Yes	12 (5.85)	7 (5.26)	5 (6.94)		
Family genetic history, *n* (%)	No	191 (93.17)	122 (91.73)	69 (95.83)	1.24	0.266
	Yes	14 (6.83)	11 (8.27)	3 (4.17)		
MDS-UPDRS score, (mean ± SD)		28.12 ± 20.15	20.98 ± 16.19	41.29 ± 20.10	−7.33	<0.001
UPDRSI sore, (mean ± SD)		3.80 ± 2.99	3.13 ± 2.82	5.04 ± 2.88	−4.58	<0.001
UPDRSII sore, (mean ± SD)		8.30 ± 6.96	6.14 ± 5.94	12.29 ± 6.94	−6.33	<0.001
UPDRSIII sore, (mean ± SD)		13.11 ± 10.32	9.60 ± 7.93	19.60 ± 11.05	−6.74	<0.001
UPDRSIV sore, (mean ± SD)		2.91 ± 2.76	2.12 ± 2.41	4.36 ± 2.77	−5.74	<0.001
HAMD, (mean ± SD)		8.05 ± 5.03	7.22 ± 4.54	9.58 ± 5.49	−3.28	0.001
HAMA, (mean ± SD)		8.34 ± 4.77	7.41 ± 4.31	10.06 ± 5.09	−3.91	<0.001
Educational years, (mean ± SD)		10.53 ± 3.96	10.86 ± 3.92	9.93 ± 3.96	1.6	0.111
MOCA, (mean ± SD)		20.38 ± 4.92	21.44 ± 4.73	18.42 ± 4.64	4.38	<0.001
Execution, (mean ± SD)		3.00 ± 1.28	3.26 ± 1.24	2.53 ± 1.21	4.02	<0.001
Naming, (mean ± SD)		2.66 ± 0.58	2.74 ± 0.49	2.50 ± 0.69	2.66	0.009
Memory, (mean ± SD)		2.32 ± 1.58	2.48 ± 1.62	2.01 ± 1.48	2.03	0.044
Attention, (mean ± SD)		4.31 ± 1.27	4.51 ± 1.24	3.94 ± 1.25	3.1	0.002
Language, (mean ± SD)		1.80 ± 0.87	1.95 ± 0.87	1.51 ± 0.80	3.56	<0.001
Abstract, (mean ± SD)		1.21 ± 0.72	1.29 ± 0.72	1.08 ± 0.70	1.93	0.055
Orientation, (mean ± SD)		5.08 ± 1.02	5.21 ± 0.96	4.83 ± 1.08	2.56	0.011
Weight loss, *n* (%)	No	180 (87.80)	125 (93.98)	55 (76.39)	13.51	<0.001
	Yes	25 (12.20)	8 (6.02)	17 (23.61)		
Weakness, *n* (%)	No	142 (69.27)	119 (89.47)	23 (31.94)	72.63	<0.001
	Yes	63 (30.73)	14 (10.53)	49 (68.06)		
Exhaustion, *n* (%)	No	109 (53.17)	105 (78.95)	4 (5.56)	101.05	<0.001
	Yes	96 (46.83)	28 (21.05)	68 (94.44)		
Slowness, *n* (%)	No	108 (52.68)	104 (78.20)	4 (5.56)	98.88	<0.001
	Yes	97 (47.32)	29 (21.80)	68 (94.44)		
Low activity, *n* (%)	No	162 (79.02)	126 (94.74)	36 (50.00)	56.4	<0.001
	Yes	43 (20.98)	7 (5.26)	36 (50.00)		

Modified H&Y, The modified Hoehn and Yahr Staging Scale; UPDRS, Unified Parkinson’s Disease Rating Scale; LEDD, Levodopa equivalent daily dose; CHD, Coronary Heart Disease; MOCA, Montreal Cognitive Assessment; HAMD, Hamilton Depression Rating Scale; HAMA, Hamilton Anxiety Rating Scale; *p*※ values for two-group comparison: Non-frailty vs. Frailty.

Among these patients, 133 were classified as non-frail (including 66 with pre-frailty) and 72 as frail. Clinical data were compared between the non-frail group (mean age: 62.92 ± 9.69 years; 59 females) and the frail group (mean age: 68.13 ± 8.44 years; 45 females).

No significant differences were observed between the two groups in disease duration (*p* = 0.723), PD subtypes (tremor-dominant, rigidity-dominant, or mixed type; *p* = 0.268), levodopa equivalent daily dose (LEDD; *p* = 0.354), or educational level (*p* = 0.111). Similarly, no significant group differences were found in comorbidities, including diabetes mellitus (*p* = 0.369), hypertension (*p* = 0.897), coronary heart disease (*p* = 0.304), stroke (*p* = 0.438), number of chronic diseases (*p* = 0.960), or lifestyle factors such as smoking (*p* = 0.166), tea consumption (*p* = 0.735), coffee consumption (*p* = 0.948), toxin exposure (*p* = 0.624), and family history of hereditary disease (*p* = 0.266).

In contrast, significant differences were identified in gender (*p* = 0.013), age (*p* < 0.001), and disease severity, as indicated by the MDS-UPDRS total score (*p* < 0.001), Modified Hoehn and Yahr (H&Y) stage (*p* < 0.001), and early versus advanced PD stage (*p* < 0.001). Specifically, female gender, older age, and greater disease severity were associated with a higher likelihood of frailty. Additionally, frail patients exhibited significant cognitive impairment (*p* < 0.001). Statistical differences were also observed in alcohol consumption history (*p* = 0.010), as well as in Hamilton Depression Rating Scale (HAMD; *p* = 0.001) and Hamilton Anxiety Rating Scale (HAMA) scores (*p* < 0.001), indicating more severe depressive and anxiety symptoms among frail patients.

**Figure 1:**
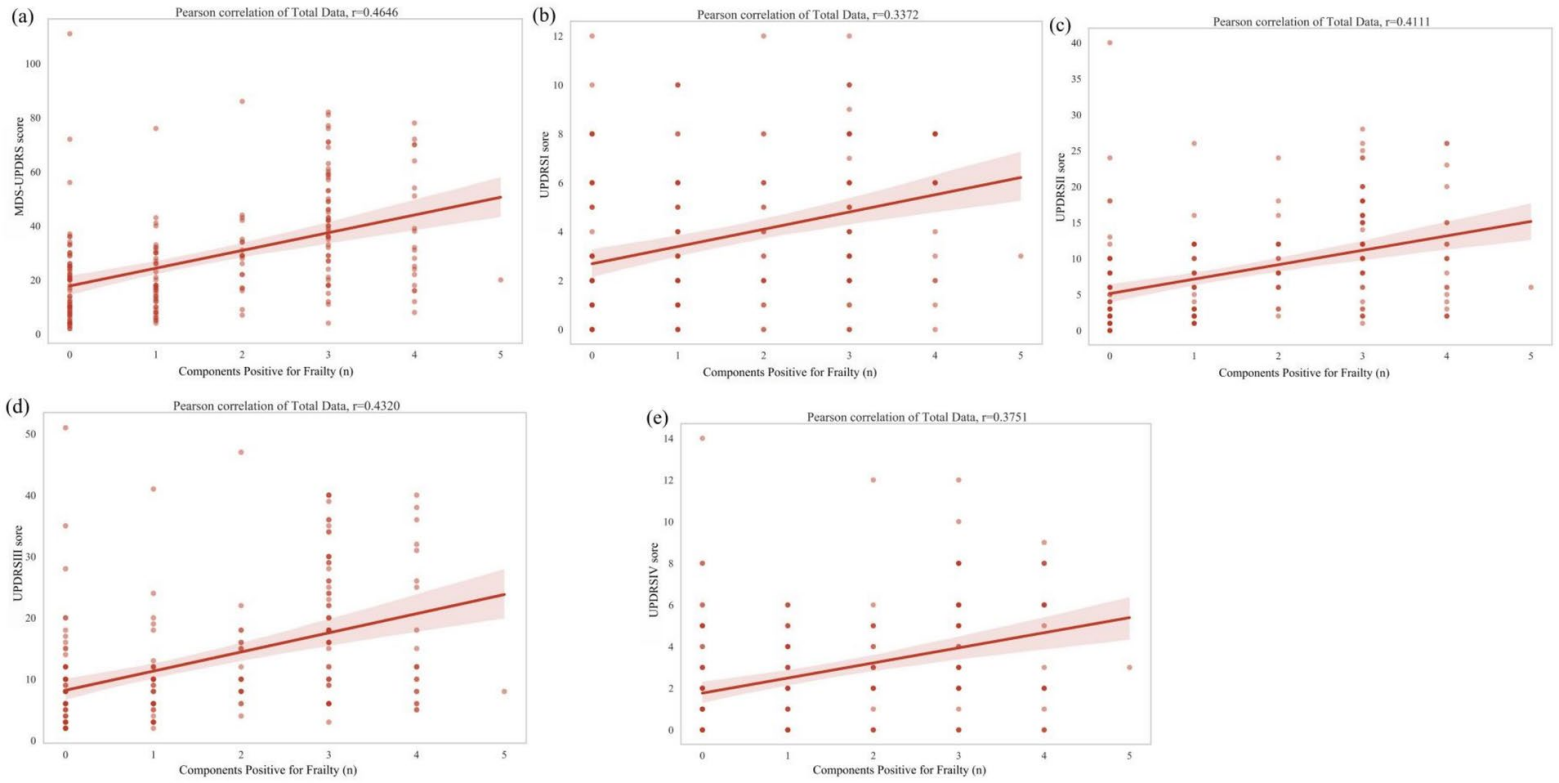
Relationship between severity of Parkinson’s disease (MDS-UPDRS) and the number of diagnostic components scoring positive for frailty.

As shown in Figure 1, a correlation analysis was performed between the number of positive frailty assessment criteria and the MDS-UPDRS scores. The results demonstrated that the number of positive frailty criteria was positively correlated with both the total MDS-UPDRS score and each subscore (Parts I–IV), with correlation coefficients (r) of 0.4646, 0.3372, 0.4111, 0.4320, and 0.3751, respectively.

**Figure 2:**
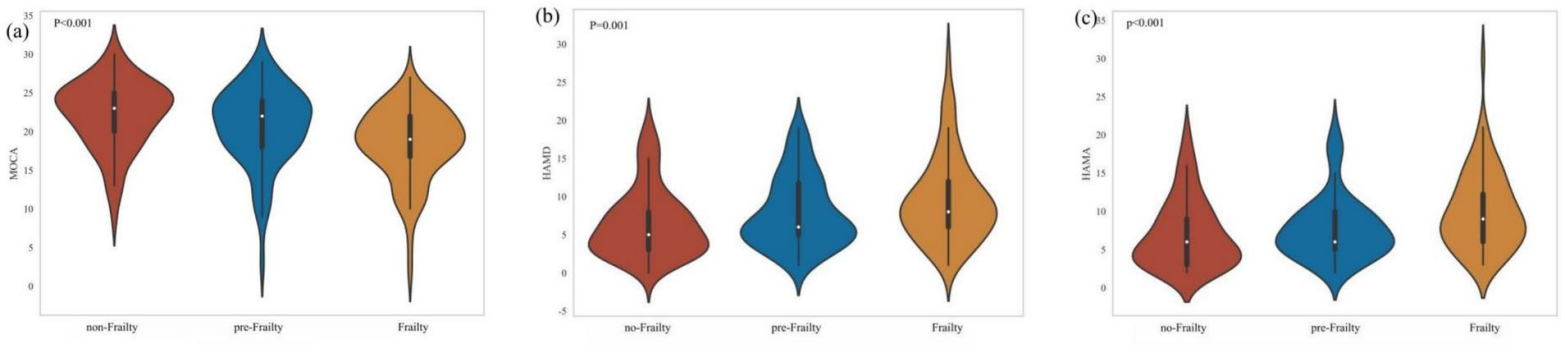
ANOVA test for MOCA, HAMD and HAMA among no-frailty, pre-frailty, and frailty groups.

As presented in Figure 2, patients in the pre-frail and frail stages exhibited progressively worse cognitive function (*p* < 0.001), along with increasing severity of depression (*p* = 0.001) and anxiety (*p* < 0.001), compared with the non-frail group.

**Figure 3:**
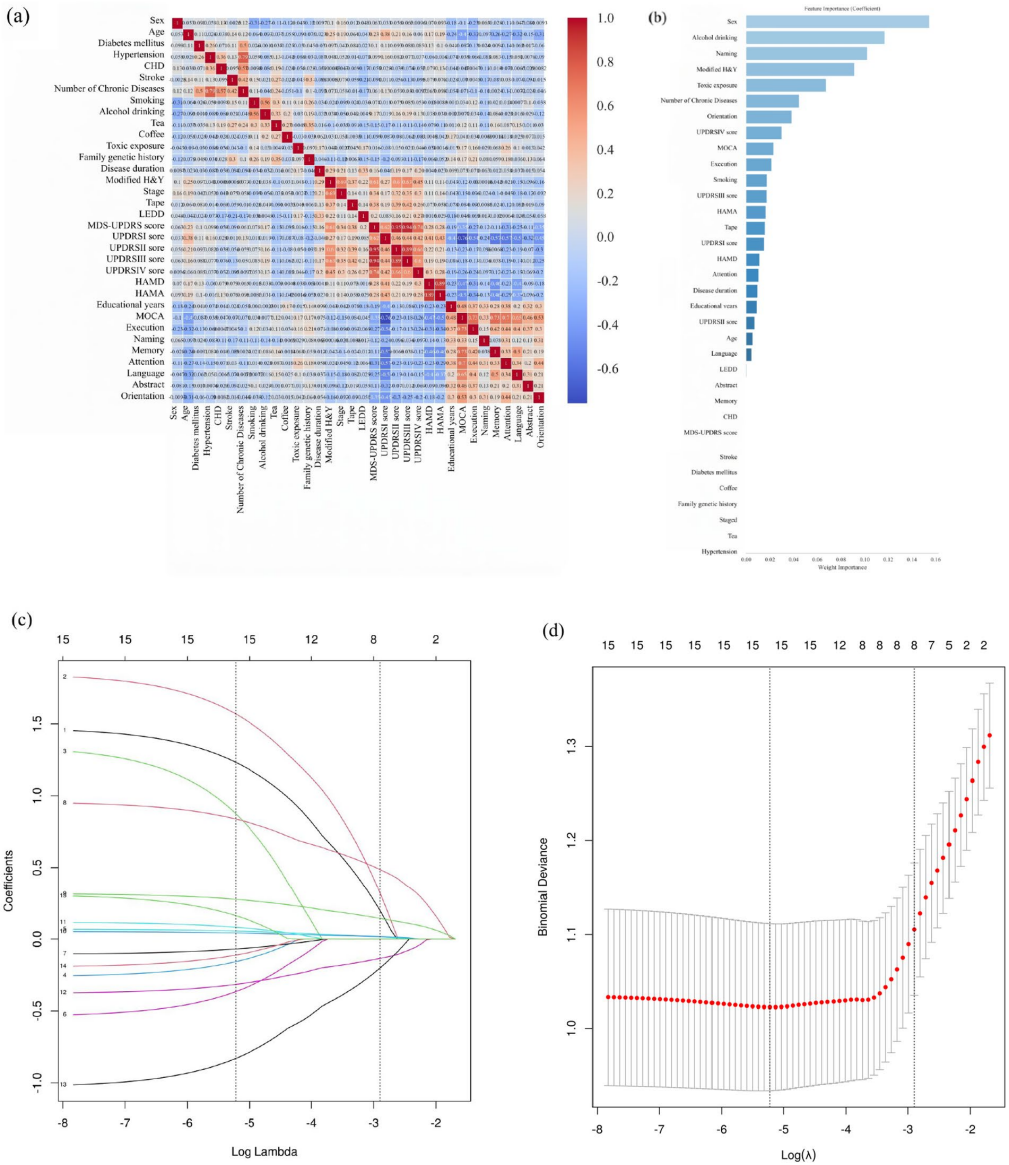
Screening of independent risk factor using Spearman correlation analysis and LASSO regression algorithm. **(a)** Spearman correlation analysis was conducted between each pair of variables, where a Spearman’s rho coefficient greater than 0.5 indicates a strong correlation. **(b)** Impact factor importance analysis. **(c,d)** LASSO regression prevents model overfitting and screens for key variables.

As illustrated in Figure 3 and Supplementary Table 1, independent risk factors for frailty were screened from the 34 collected variables. Based on Spearman’s rank correlation analysis (Figure 3a) and influence factor importance analysis (Figure 3b), alcohol consumption and smoking were found to be highly correlated (r = 0.56); however, given that alcohol consumption demonstrated greater importance, smoking was excluded. Following this approach, 15 potential risk factors were identified, including gender, age, number of chronic diseases, alcohol consumption, history of toxin exposure, disease duration, modified H&Y stage, PD subtype, UPDRS IV score, HAMA score, years of education, executive function, naming ability, attention, and orientation.

Subsequent LASSO regression analysis (Supplementary Tables 1a,b; Figures 3c,d) identified eight independent risk factors significantly associated with frailty in PD: gender, age, alcohol consumption, modified H&Y stage, UPDRS IV score, HAMA score, executive function, and naming ability.

**Figure 4:**
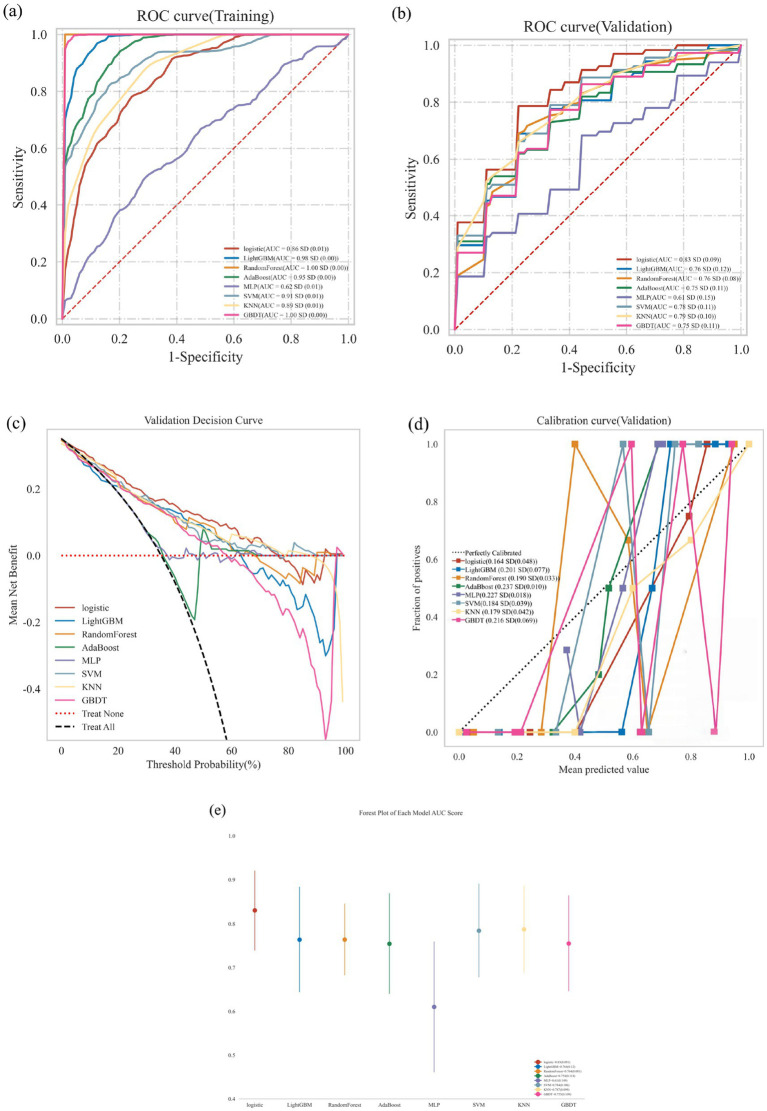
Classification results of multiple models (including training set and validation set). Comprehensive analysis of classification models. **(a)** Receiver operating characteristic (ROC) curves and area under the curve (AUC) for the training set; **(b)** ROC curves and AUC for the validation set. PD patients were sampled 10 times using a 7:3 split ratio. **(c)** Decision curve analysis (DCA) for the validation set, where the black dashed line assumes that all patients have frailty, and the red dashed line assumes no patients have frailty. The remaining solid lines represent different models. **(d)** Calibration curves for the validation set, where the x-axis represents the mean predicted probability, and the y-axis represents the actual probability of the event. The dashed diagonal line serves as the reference line, while the remaining smoothed solid lines represent the fitted lines for different models. A fitted line closer to the reference line, with smaller values in parentheses, indicates greater predictive accuracy of the model. **(e)** Forest plot comparing the AUC scores and confidence intervals of various models.

As shown in Table 2 and Figure 4, the eight identified independent risk factors for frailty were incorporated into eight machine learning algorithms: Logistic Regression (LR), Light Gradient Boosting Machine (LGBM), Random Forest (RF), Adaptive Boosting (AdaBoost), Multi-Layer Perceptron (MLP), Support Vector Machine (SVM), K-Nearest Neighbors (KNN), and Gradient Boosting Decision Tree (GBDT). Each model was trained and evaluated over ten repeated iterations, and performance was assessed using the Area Under the Receiver Operating Characteristic Curve (AUC) metric.

**Table 2:** Classification results of multiple models (including training set and validation set).

Classification model	AUC (SD)	Cutoff (SD)	Accuracy (SD)	Sensitivity (SD)	Specificity (SD)	Positive predictive value (SD)	Negative predictive value (SD)	F1 score (SD)	Kappa (SD)
Training set									
LR	0.86 (0.01)	0.31 (0.06)	0.76 (0.03)	0.84 (0.06)	0.72 (0.07)	0.63 (0.04)	0.90 (0.03)	0.71 (0.01)	0.52 (0.03)
LGBM	0.98 (0.00)	0.39 (0.05)	0.92 (0.02)	0.97 (0.03)	0.90 (0.03)	0.84 (0.04)	0.98 (0.01)	0.90 (0.02)	0.84 (0.03)
RF	1.00 (0.00)	0.52 (0.03)	1.00 (0.00)	1.00 (0.00)	1.00 (0.00)	1.00 (0.00)	1.00 (0.00)	1.00 (0.00)	1.00 (0.00)
AdaBoost	0.95 (0.00)	0.49 (0.00)	0.86 (0.02)	0.95 (0.04)	0.82 (0.05)	0.74 (0.04)	0.97 (0.02)	0.83 (0.02)	0.72 (0.03)
MLP	0.62 (0.01)	0.43 (0.01)	0.64 (0.02)	0.53 (0.08)	0.70 (0.08)	0.49 (0.03)	0.73 (0.02)	0.51 (0.03)	0.22 (0.03)
SVM	0.91 (0.01)	0.31 (0.04)	0.84 (0.01)	0.84 (0.04)	0.84 (0.04)	0.74 (0.03)	0.91 (0.02)	0.78 (0.01)	0.66 (0.02)
KNN	0.89 (0.01)	0.43 (0.07)	0.77 (0.02)	0.87 (0.08)	0.72 (0.07)	0.64 (0.06)	0.92 (0.03)	0.73 (0.01)	0.55 (0.02)
GBDT	1.00 (0.00)	0.44 (0.05)	0.98 (0.00)	0.99 (0.01)	0.98 (0.01)	0.96 (0.02)	0.99 (0.01)	0.98 (0.01)	0.96 (0.01)
Validation set									
LR	0.83 (0.09)	0.31 (0.06)	0.72 (0.09)	0.76 (0.19)	0.69 (0.14)	0.59 (0.11)	0.86 (0.10)	0.65 (0.12)	0.42 (0.17)
LGBM	0.76 (0.12)	0.39 (0.05)	0.73 (0.11)	0.65 (0.20)	0.77 (0.13)	0.63 (0.17)	0.81 (0.10)	0.62 (0.14)	0.41 (0.22)
RF	0.76 (0.08)	0.52 (0.03)	0.74 (0.06)	0.56 (0.21)	0.84 (0.08)	0.66 (0.14)	0.79 (0.08)	0.58 (0.15)	0.40 (0.16)
AdaBoost	0.75 (0.11)	0.49 (0.00)	0.72 (0.07)	0.75 (0.20)	0.71 (0.10)	0.58 (0.08)	0.85 (0.10)	0.64 (0.11)	0.42 (0.16)
MLP	0.61 (0.15)	0.43 (0.01)	0.58 (0.14)	0.44 (0.24)	0.65 (0.22)	NaN(NaN)	0.68 (0.11)	NaN(NaN)	0.10 (0.26)
SVM	0.78 (0.11)	0.31 (0.04)	0.70 (0.09)	0.68 (0.19)	0.71 (0.12)	0.57 (0.12)	0.81 (0.10)	0.61 (0.12)	0.37 (0.18)
KNN	0.79 (0.10)	0.43 (0.07)	0.67 (0.10)	0.69 (0.28)	0.65 (0.17)	NaN(NaN)	0.82 (0.13)	NaN(NaN)	0.31 (0.22)
GBDT	0.75 (0.11)	0.44 (0.05)	0.70 (0.13)	0.54 (0.20)	0.78 (0.13)	0.59 (0.20)	0.76 (0.10)	0.55 (0.18)	0.33 (0.27)

LR, Logistic Regression; LGBM, Light Gradient Boosting Machine; RF, Random Forest; AdaBoost, Adaptive Boosting; MLP, Multilayer Perceptron; SVM, Support Vector Classifier; KNN, K-Nearest Neighbors; GBDT, Gradient Boosting Decision Tree.

In the training dataset, the AUC values were as follows: LR (0.86), LGBM (0.98), RF (1.00), AdaBoost (0.95), MLP (0.62), SVM (0.91), KNN (0.89), and GBDT (1.00). In the validation dataset, AUC values were: LR (0.83), LGBM (0.76), RF (0.76), AdaBoost (0.75), MLP (0.61), SVM (0.78), KNN (0.79), and GBDT (0.75) (Table 2; Figures 4a,b). Although AUC reflects predictive accuracy, it does not fully capture clinical applicability or the comparative superiority of models.

Therefore, additional evaluations were conducted, including Decision Curve Analysis (DCA), calibration curves, and forest plots. DCA demonstrated that LR provided superior clinical utility compared with other algorithms (Figure 4c). Calibration curves further confirmed that LR achieved the highest predictive accuracy (calibration error = 0.164; Figure 4d). Forest plot analysis revealed significant differences in AUC scores among the models, with LR consistently achieving the highest AUC value (Figure 4e). Collectively, these results indicate that Logistic Regression is the optimal model for predicting frailty in PD patients.

**Figure 5:**
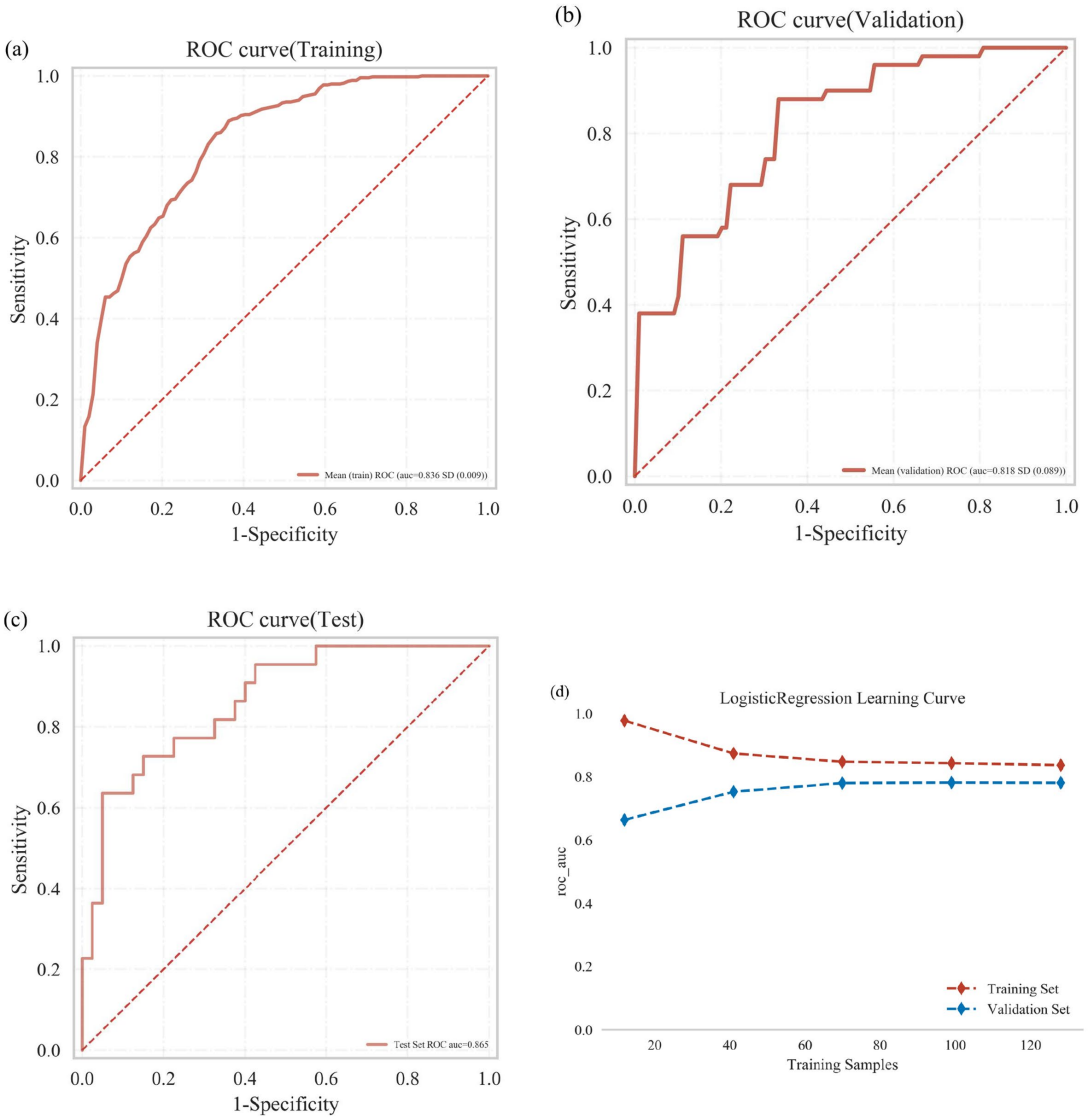
Logistic regression model training, validation, and testing. **(a)** Receiver operating characteristic (ROC) curve and area under the curve (AUC) for the training set. **(b)** ROC and AUC for the validation set. **(c)** ROC and AUC for the test set, based on results from 30% of PD patients. **(d)** Learning curves, with the red dashed line representing the training set and the blue dashed line representing the validation set.

As shown in Figure 5, logistic regression (LR) analysis combined with 10-fold cross-validation was performed on the training dataset. The model achieved an average area under the receiver operating characteristic curve (AUC) of 0.836 for the training set, 0.818 for the validation set, and 0.865 for the test set (Figures 5a–c). The AUC values across the three datasets were consistently stable around 0.84, indicating robust predictive performance of the model. A model is generally considered well-fitted when the AUC of the validation set does not differ from that of the test set by more than 10%. The learning curve demonstrated strong fitting capacity and high stability for both the training and validation sets (Figure 5d).

**Figure 6:**
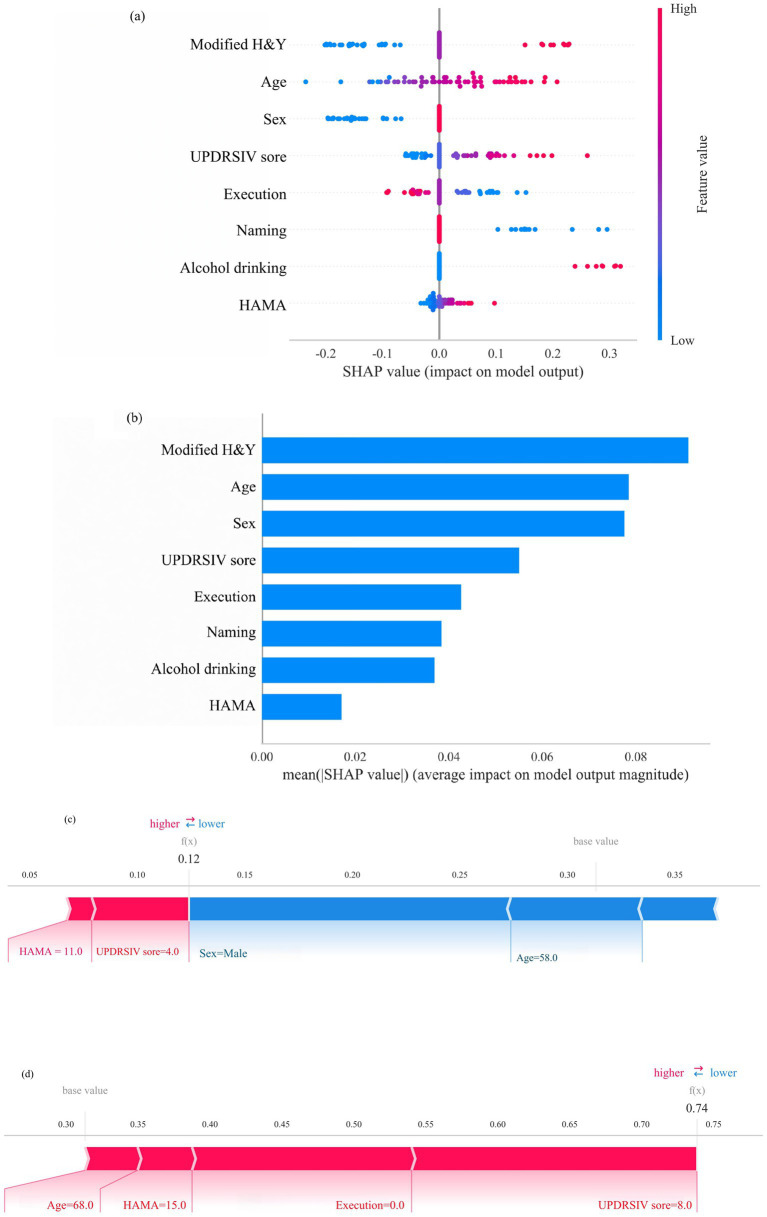
SHAP model interpretation. **(a)** Feature attributions in SHAP. Each line represents a feature, with the x axis indicating SHAP values. Red dots correspond to high feature values, while blue dots indicate low feature values. **(b)** Feature importance ranking represented by SHAP. The matrix plot illustrates the significance of each covariate in developing the final predictive model. **(c)** Interpretability model for a PD patient without frailty and **(d)** a PD patient with frailty. The numbers below f(x) represent the predicted probability values, while the baseline value reflects the prediction without any input to the model. Red features indicate increased risk, whereas blue features signify reduced risk. The length of the arrows provides a visual representation of the degree to which predictions are influenced; longer arrows indicate greater impact.

As illustrated in Figure 6, the SHapley Additive exPlanations (SHAP) method was applied to enhance the interpretability of the model and to visualize the contribution of each variable to frailty prediction in PD. Figure 6a displays the eight predictive features used in the model. Each line represents the feature attribution for all patients, with red dots indicating higher-risk feature values and blue dots indicating lower-risk feature values—both associated with variations in frailty risk among PD patients. Figure 6b ranks the eight independent risk factors according to their mean absolute SHAP values, with the SHAP value on the x-axis representing their relative importance to the model’s prediction.

Furthermore, two representative cases were selected to illustrate the model’s interpretability. One case involved a non-frail PD patient, showing a low predicted probability of frailty [f(x) = 0.12, Figure 6c]. The other case represented a frail PD patient, with a high predicted probability of frailty [f(x) = 0.74, Figure 6d].

## 4 Discussion

In this study, we developed a simple auxiliary tool for early frailty screening in patients with Parkinson’s disease (PD) using clinical data and machine learning methods. To the best of our knowledge, this represents the first report worldwide describing a machine learning–based model for identifying frailty in PD. Moreover, our findings revealed that as PD patients progress from the pre-frail to the frail stage, their cognitive impairment, depressive symptoms, and anxiety levels become progressively more severe.

The pathophysiological overlap between PD and frailty is driven by aging-related chronic inflammation and oxidative stress, which activate the immune system and impair neurohumoral regulation and energy balance. This leads to multisystem dysfunction—affecting the musculoskeletal, endocrine, nervous, and other systems—a process also implicated in neurodegeneration. Therefore, frailty in prodromal PD likely stems from a combination of direct PD-related pathology and comorbid conditions that synergistically worsen overall health (Blesa et al., 2022; Dzięgielewska-Gęsiak and Muc-Wierzgoń, 2023). Consistent with these findings, our study showed that female sex, older age, and greater PD severity were significantly associated with frailty. Similar associations have been reported in prior studies (Fried et al., 2001; Ahmed et al., 2008; Clegg et al., 2013; Smith et al., 2021). These factors likely contribute to sarcopenia and nutritional decline, which are key determinants of frailty. Specifically, they can lead to reduced grip strength, weight loss, and decreased physical activity, all of which correspond to positive results in multiple frailty assessment criteria (Muscaritoli et al., 2010). Together, these mechanisms may accelerate the development and progression of frailty in PD patients. In this study, we found that female patients with PD were more likely to develop frailty. This finding is physiologically plausible, as women generally have lower lean body mass and muscle strength compared with men (Fried et al., 2001). In addition, previous research has shown that men are at greater risk of sudden death, whereas women exhibit a more gradual decline in physical function and mortality over time. This slower decline may lead to progressive physical weakness, resulting in a higher prevalence of frailty-related features among women (Puts et al., 2005). Furthermore, women typically have a longer life expectancy, and because frailty severity increases with advancing age, this may further contribute to the observed sex differences. These findings are consistent with prior reports (Morley, 2008; Collard et al., 2012). Our results also indicated that alcohol consumption was associated with frailty among PD patients. Previous studies have demonstrated a dose-dependent inverse relationship between alcohol intake and the risk of developing PD (Campdelacreu, 2014; Mitchell et al., 2022). Unlike the aforementioned studies, several reports have indicated that alcohol consumption is associated with an increased risk of PD (Peng et al., 2020). Chronic or excessive alcohol intake may aggravate neurodegenerative processes through multiple mechanisms, including oxidative stress, mitochondrial dysfunction, neuroinflammation, and apoptosis (Kamal et al., 2020). In the field of frailty research, previous studies have demonstrated that alcohol consumers are more likely to develop frailty compared with lifelong abstainers (Xia et al., 2024). Notably, heavy alcohol consumption during midlife (defined as a weekly intake exceeding 196 grams) has been identified as a significant risk factor for frailty in later life (Strandberg et al., 2018). A research team from the University of East Anglia (UK) analyzed data from approximately 200,000 participants aged 37–73 years in the UK Biobank and found that individuals with the highest alcohol intake exhibited lower skeletal muscle mass. These findings suggest that habitual heavy drinking in midlife and early older age may accelerate muscle loss, which is closely associated with the onset and progression of frailty (Skinner et al., 2023). Our study revealed a positive association between alcohol consumption and frailty in PD, which was further confirmed by the machine learning model analysis. Although this correlation may be influenced by factors such as drinking volume and duration, our findings suggest that alcohol consumption may contribute to the progression of frailty in individuals with PD.

In this study, PD patients with frailty exhibited significantly higher scores on the Movement Disorder Society–Unified Parkinson’s Disease Rating Scale (MDS-UPDRS). Furthermore, the MDS-UPDRS total score was positively correlated with the number of positive frailty assessment criteria, indicating a close association between disease severity and frailty. This finding underscores the dynamic interaction between PD progression and frailty, which has also been documented in previous studies (Smith et al., 2021). The MDS-UPDRS is an internationally recognized instrument for the comprehensive evaluation of symptom severity, functional status, and quality of life in PD patients. Among its subcomponents, our study identified the UPDRS Part IV score—reflecting complications related to PD treatment—as having a particularly strong influence on frailty. Previous research has shown that changes in motor function can contribute to frailty development, while higher levels of physical activity may mitigate frailty progression by modulating pathophysiological pathways, including the reduction of systemic inflammatory markers (Aguirre and Villareal, 2015; Cerqueira et al., 2019). Taken together, these findings suggest that disease severity, functional impairment, and treatment-related complications play pivotal roles in the onset and progression of frailty in PD patients, further emphasizing the need for comprehensive management strategies addressing both motor and non-motor domains.

Previous studies have consistently demonstrated that cognitive impairment is a common non-motor symptom in PD (Agosta et al., 2013; Pereira et al., 2014). The co-occurrence of frailty and cognitive impairment is a powerful predictor of clinically significant adverse outcomes in patients (Chen et al., 2022). This interaction may be partly explained by the close association between cognitive function and gait performance. Cognitive domains such as executive function, attention, visuospatial ability, and memory have been shown to influence walking speed and mobility (Amboni et al., 2013; Lin et al., 2019). Cognitive decline in PD is thought to result from the combined effects of endocrine dysregulation, dopaminergic neuronal loss, cholinergic dysfunction, and structural alterations in cortical and subcortical regions (Höglinger et al., 2004; Delgado-Alvarado et al., 2016).β-amyloid deposition, known for its neurotoxic effects, particularly in the left angular gyrus and occipital cortex, has been linked to impaired cognitive function (Stevens et al., 2022). Frailty in PD has a particularly strong impact on memory impairment (Lin et al., 2019), and our findings further elucidate potential neurobiological mechanisms underlying this association. The cognitive effects of frailty appear to be primarily localized to the temporal, parietal, and occipital lobes, regions that are crucial to cholinergic neurotransmission (van der Zee et al., 2022). The posterior cortex, which receives cholinergic projections from the basal ganglia, often shows evidence of cholinergic denervation in PD (Hilker et al., 2005).

In the machine learning model of this study, executive function and naming ability emerged as key contributors to the development of frailty in PD patients. Visual stimuli are transmitted from the occipital lobe to the temporal lobe, which subsequently activates the limbic system and modulates reward processing in the caudate nucleus via the dopaminergic pathway. Dysfunction along this critical pathway has been implicated in executive impairment (Grill et al., 2021; Nishimura et al., 2022). Executive dysfunction is particularly pronounced in PD patients with frailty (Muslimovic et al., 2005), suggesting that frailty may exacerbate executive deficits through this neural circuit.

Naming ability, which is closely associated with the temporal lobe, depends on the integration of semantic and visual information to support lexical retrieval and word production. Previous studies have shown that older PD patients with mild cognitive impairment (MCI) and concomitant frailty tend to progress to dementia over a relatively short period. Even when the degree of cognitive impairment is comparable between PD-MCI converters and non-converters, cortical thinning of the middle temporal gyrus has been observed in the former group (Gasca-Salas et al., 2019). The right inferior temporal gyrus (Temporal_Inf_R), primarily involved in language comprehension, is particularly susceptible to structural damage. Such alterations may negatively affect the naming ability of frail PD patients (Carvalho de Abreu et al., 2023).

Approximately 20–30% of patients with Parkinson’s disease (PD) experience emotional disorders like anxiety and depression, with depressive symptoms often emerging prior to clinical diagnosis. Notably, PD patients with comorbid frailty are particularly susceptible to these symptoms. Furthermore, in the elderly, depression—alongside impaired cardiac function—has been identified as a significant risk factor for frailty, a finding that is consistent with the results of this study (Schrag et al., 2002; Alonso et al., 2009; Bloem et al., 2021; Bakhtiari et al., 2024; Tang et al., 2025). Because emotional disturbances often overlap with the cognitive and motor symptoms of PD, they are frequently underrecognized or misdiagnosed (Weintraub et al., 2003; Marsh et al., 2006). Anxiety and depression commonly coexist and can be easily confounded with cognitive impairment. Previous studies have demonstrated a strong association between depression and physical frailty in the elderly, with shared clinical manifestations including sadness, apathy, cognitive decline, and physical deterioration. Physical frailty increases the risk of depression, while depression may, in turn, accelerate frailty progression by diminishing self-care ability and reducing engagement in health-promoting behaviors (Silva et al., 2025).

In the present study, scores on the Hamilton Anxiety Rating Scale (HAMA) and the 17-item Hamilton Depression Rating Scale (HAMD) were positively correlated with the progression from non-frailty to pre-frailty and ultimately to frailty among PD patients. These findings suggest that the development of frailty exacerbates anxiety and depressive symptoms in PD. The comorbidity of PD and frailty likely reflects shared neuropathophysiological mechanisms. For instance, depression has been linked to more severe motor dysfunction and decreased dopamine transporter (DAT) activity (Goetz, 2010; Nègre-Pagès et al., 2010).

Given this dynamic interaction, early detection and timely intervention for anxiety and depression are essential, particularly during the non-frail or pre-frail stages of PD. Targeted management may significantly improve patients’ overall condition and quality of life. Elderly patients often focus primarily on somatic symptoms such as fatigue, loss of energy, pain, decreased libido, functional decline, and sleep disturbances. Evidence suggests that preventive strategies can delay or mitigate frailty progression while enhancing cognitive and emotional well-being through various physical, cognitive, and social activities (Pinto et al., 2024). Furthermore, individualized multimodal approaches—encompassing cognitive behavioral therapy, psychoeducation, social and psychological support, and pharmacological treatment—should be considered to optimize outcomes in PD patients with frailty (Ray and Agarwal, 2020; Zhang et al., 2020).

The results of this study demonstrated that, through baseline comparison, Spearman correlation analysis, influence factor importance analysis, and LASSO regression analysis, eight independent clinical variables were identified from a pool of 34 features. These variables included gender, age, alcohol consumption, modified Hoehn-Yahr (H&Y) stage, UPDRS Part IV score, HAMA score, executive function, and naming ability. Detailed analyses and discussions of these risk factors have been provided above. Collectively, these eight variables were determined to be significant independent predictors of frailty in PD patients and can therefore be utilized for frailty risk assessment.

We acknowledge that some expected factors, such as LEDD and UPDRS parts I–III, were not independent predictors in our final model. We have added hypotheses to explain this. The effect of LEDD on frailty may be indirect and better captured by overall disease severity measures like H&Y stage. Although smoking is an established risk factor for Parkinson’s disease (PD), our univariate analysis revealed no significant association with frailty. This is likely attributable to the exceptionally low smoking rate in our cohort, which resulted in limited statistical power. Consequently, smoking was not considered as an independent risk factor in the final model. Given that our cohort was restricted to early-to-mid-stage PD, the range of these variables was likely constrained, thereby reducing their utility in discriminating frailty. Consequently, a larger sample might be necessary to detect the subtler associations of these factors.

These variables were incorporated into eight machine learning algorithms, including Logistic Regression (LR), Light Gradient Boosting Machine (LGBM), Random Forest (RF), Adaptive Boosting (AdaBoost), Multi-Layer Perceptron (MLP), Support Vector Machine (SVM), K-Nearest Neighbors (KNN), and Gradient Boosting Decision Tree (GBDT). Among these models, Logistic Regression demonstrated superior performance for classification modeling and was the most accurate method for predicting frailty in PD patients.

Logistic Regression offers several advantages that account for its optimal performance in this study. It is a classic linear discriminant model widely used for binary classification tasks. By mapping the continuous output of linear regression to the interval [0,1] via the Sigmoid function, LR produces interpretable probability estimates for sample classification. Its core strengths include high interpretability, efficient training and prediction, low data and computational requirements, strong generalization ability (with low risk of overfitting), and adaptability to diverse clinical scenarios. These characteristics make LR particularly well suited for early frailty identification and prediction model development in PD patients.

Previous studies have confirmed the robustness of Logistic Regression for clinical prediction tasks, especially in settings with low-dimensional binary outcomes, such as epidemiological, health service, and neuropsychological research (Pavlou et al., 2016; Christodoulou et al., 2019; Ariza et al., 2022). Moreover, LR has demonstrated strong performance in integrating clinical and biomechanical features, maintaining a favorable balance between accuracy and interpretability (Rijnen et al., 2020).

Compared with Logistic Regression, the Light Gradient Boosting Machine (LightGBM) is a gradient boosting decision tree algorithm that accelerates training by optimizing gradients over data instances. It offers fast training and strong predictive performance, but is more sensitive to outliers. The Random Forest (RF) model, another ensemble decision tree–based algorithm, enhances prediction accuracy by aggregating results from multiple trees and is recognized for its robustness and generalizability, though it demands greater computational resources.

Adaptive Boosting (AdaBoost) is an ensemble algorithm based on weighted voting, which improves performance by iteratively adjusting the weights of training samples. It is robust to outliers but may be prone to overfitting in certain cases. The K-Nearest Neighbors (KNN) classifier is a lazy learning model that does not require explicit training; however, it suffers from low prediction efficiency and is sensitive to high-dimensional data. The Support Vector Machine (SVM) is a structural risk minimization model with strong generalization capability, but it has low training efficiency, is sensitive to kernel function selection, and is prone to performance degradation in cases of class imbalance.

The Multi-Layer Perceptron (MLP), a fundamental deep learning model, is capable of capturing complex nonlinear relationships, but is hindered by high training costs, low interpretability, sensitivity to data preprocessing, and susceptibility to overfitting. The Gradient Boosting Decision Tree (GBDT) is a boosting-based ensemble learning framework with strong predictive ability, but it is sensitive to outliers, has limited parallelism, requires complex hyperparameter tuning, and generally performs poorly on high-dimensional sparse datasets.

Although this study evaluated these more complex models, their performance was inferior to that of Logistic Regression. This may be attributable to the relatively small sample size or the simplicity of the feature space. These findings underscore the importance of selecting machine learning models based not only on algorithmic complexity but also on dataset characteristics and clinical applicability (Vergunst et al., 2019; de Toro-Martín et al., 2018). Machine learning models can be developed either individually or in combination as ensemble models, enabling the integration of multiple simpler models to construct a more powerful predictive system. Such approaches are widely used to identify complex patterns—such as nonlinear correlations, interactions, latent dimensions, or subgroups—within high-dimensional data structures (Jiang et al., 2020).

In this study, we developed an effective machine learning–based prediction model for frailty in Parkinson’s disease (PD), with Logistic Regression emerging as the optimal algorithm. This model was built upon readily available clinical data, overcoming several limitations of previous studies. For instance, existing approaches often rely on wearable devices incorporating sensor technology or on biomarker data to predict frailty status. However, the high costs of wearable devices and the challenges associated with collecting biomarker data limit the feasibility of large-scale implementation (Gomez-Cabrero et al., 2021; Park et al., 2021). The core advantage of this study is reflected in its comprehensive variable coverage targeting a specific PD population, its robust methodology combining LASSO regression with machine learning to enhance predictive accuracy and stability, and its high clinical translational value via an effective screening tool (logistic regression model, AUC = 0.83) that solves the practical challenge of rapid frailty identification.

The prediction models developed in this study demonstrated robust performance, supporting the feasibility of applying machine learning to frailty prediction in PD. While there is no cure for PD, frailty is modifiable (Racey et al., 2021). Therefore, early intervention for pre-frailty in PD patients, which has the potential to prevent or reverse its progression and may enhance the efficacy of symptomatic PD treatments, holds significant clinical relevance. Importantly, our findings suggest that integrating machine learning algorithms with routinely collected clinical data offers a practical, cost-effective approach for early identification of frailty in PD patients, with potential for broad clinical application and population-level screening.

## 5 Conclusion

In summary, this study demonstrated that female gender, advanced age, alcohol consumption, and greater disease severity are significant risk factors for frailty in patients with Parkinson’s disease (PD). PD patients with frailty exhibited higher MDS-UPDRS scores, more pronounced cognitive impairment, and more severe symptoms of depression and anxiety. By identifying independent risk factors and systematically comparing the performance of multiple machine learning algorithms, we developed a robust prediction model capable of facilitating the early detection of frailty in PD patients.

The integration of routinely collected clinical data with machine learning techniques represents a promising and practical approach to enhancing the management and prognosis of PD. This method offers potential for early intervention and individualized patient care. Future studies should aim to validate these findings in larger, multicenter cohorts and refine the model to establish a comprehensive, clinically applicable diagnostic tool for predicting frailty and related cognitive complications in PD.

## 6 Limitations

Although this study provides valuable insights, several limitations should be acknowledged. First, while the sample size was sufficient for preliminary analysis, it may not fully represent the broader population of PD patients, particularly those at different disease stages. Larger-scale, multicenter studies are needed to validate these findings and assess their generalizability.

In clinical practice, frailty-related factors often coexist rather than occur in isolation, and frailty and PD interact dynamically. In this study, frailty assessment was conducted during the “on” state of PD patients; however, it was not possible to completely eliminate the interaction between the two conditions. Additionally, all participants were recruited from a single center, which may limit the representativeness of the findings. The study participants were mainly PD patients from Shenzhen and its surrounding areas. This geographical concentration has objectively limited the direct generalization of the results to populations of different ethnicities and under different healthcare systems worldwide.

To enhance the accuracy of frailty identification, future studies should incorporate larger and more diverse datasets and further optimize machine learning models. The underlying pathophysiological mechanisms and causal relationships between frailty and PD should be investigated through animal experiments and longitudinal clinical studies. Furthermore, future research should expand the range of clinical and neuropsychological variables and refine predictive models to develop an interpretable multi-classification screening tool capable of accurately identifying non-frailty, pre-frailty, and frailty stages. Such a tool would greatly facilitate early detection and intervention. We look forward to further studies that address these limitations and advance the understanding and management of frailty in PD.

A potential limitation of this study is the absence of a healthy control or non-PD comparison group. While the primary aim was to develop a frailty prediction model within the PD cohort, the inclusion of a control group could have helped contextualize the frailty findings—for instance, by clarifying whether the observed prevalence and patterns of frailty are specific to Parkinson’s disease or also present in the general aging population. Future studies should incorporate control groups to better delineate the relationship between PD and frailty.

Looking ahead, we consider “conducting prospective longitudinal studies to verify causal associations and optimize prediction models” as the most critical future direction. These studies will focus on two key objectives: (1) validating the causal relationships between established predictive factors—such as age, Hoehn & Yahr stage, and alcohol consumption—and the subsequent onset of frailty in patients with Parkinson’s disease (PD), thereby overcoming the limitations of cross-sectional studies in inferring causality; and (2) refining existing prediction models using longitudinal follow-up data to improve the accuracy of frailty risk prediction, address the shortcomings of previous studies, and render this research outlook more feasible and targeted.

## Data Availability

The original contributions presented in the study are included in the article/Supplementary material, further inquiries can be directed to the corresponding author.
