# Supplementary material for: Modeling and validation in Parkinson’s disease patients with frailty
Source: Front Neurosci. 2025 Dec 8;19:1723707. doi: 10.3389/fnins.2025.1723707 (PMC12719481; doi:10.3389/fnins.2025.1723707)
Supplement: Supplementary file 1 [file Table_1.docx]

Supplementary Table1. Eight independent risk factors were screened out using Spearman correlation analysis and LASSO regression

a. Spearman's rho coefficient

| Characteristics | Sex | Age | Alcohol drinking | Modified H&Y | UPDRSⅣ sore | HAMA | Execution | Naming |
| --- | --- | --- | --- | --- | --- | --- | --- | --- |
| Sex | 1 | 0.05 | -0.27 | 0.1 | 0.01 | 0.09 | -0.23 | 0.06 |
| Age | 0.05 | 1 | -0.09 | 0.25 | 0.06 | 0.19 | -0.32 | -0.1 |
| Alcohol drinking | -0.27 | -0.09 | 1 | 0.04 | 0.13 | 0.03 | 0.03 | -0.14 |
| Modified H&Y | 0.1 | 0.25 | 0.04 | 1 | 0.45 | 0.11 | -0.08 | 0 |
| UPDRSⅣ sore | 0.01 | 0.06 | 0.13 | 0.45 | 1 | 0.28 | -0.24 | -0.1 |
| HAMA | 0.09 | 0.19 | 0.03 | 0.11 | 0.28 | 1 | -0.34 | -0.13 |
| Execution | -0.23 | -0.32 | 0.03 | -0.08 | -0.24 | -0.34 | 1 | 0.15 |
| Naming | 0.06 | -0.1 | -0.14 | 0 | -0.1 | -0.13 | 0.15 | 1 |

b. LASSO regression

| Characteristics | Least Mean Square Error Coefficient | Standard Error Coefficient of Minimum Distance |
| --- | --- | --- |
| (Intercept) | -9.04 | -2.814 |
| Sex | 1.233 | 0.198 |
| Alcohol drinking | 1.57 | 0.321 |
| Toxic exposure | 0.876 | 0.0 |
| Tape | -0.157 | 0.0 |
| Age | 0.056 | 0.015 |
| Number of Chronic Diseases | -0.365 | 0.0 |
| Disease duration | -0.069 | 0.0 |
| Modified H&Y | 0.838 | 0.486 |
| UPDRSⅣ score | 0.277 | 0.144 |
| HAMA | 0.043 | 0.018 |
| Educational years | 0.082 | 0.0 |
| Execution | -0.315 | -0.134 |
| Naming | -0.83 | -0.201 |
| Attention | -0.111 | 0.0 |
| Orientation | 0.163 | 0.0 |
